# Direct comparison of whole heart quantifications between different retrospective and prospective gated 4D flow CMR acquisitions

**DOI:** 10.3389/fcvm.2024.1411752

**Published:** 2024-07-31

**Authors:** Kady Fischer, Leonard Grob, Louis Setz, Bernd Jung, Mario D. Neuenschwander, Christoph D. Utz, Hendrik von Tengg-Kobligk, Adrian T. Huber, Jan O. Friess, Dominik P. Guensch

**Affiliations:** ^1^ Department of Anaesthesiology and Pain Medicine, Inselspital, Bern University Hospital, University of Bern, Bern, Switzerland; ^2^ Department of Diagnostic, Interventional and Paediatric Radiology, Inselspital, Bern University Hospital, University of Bern, Bern, Switzerland; ^3^ Translational Imaging Center (TIC), Swiss Institute for Translational and Entrepreneurial Medicine, Sitem-Insel, Bern, Switzerland; ^4^ Department of Radiology and Nuclear Medicine, Lucerne Cantonal Hospital, University of Lucerne, Lucerne, Switzerland

**Keywords:** cardiovascular magnetic resonance, 4D flow, retrospective gating, prospective ECG triggering, diastolic function

## Abstract

**Introduction:**

4D flow cardiovascular magnetic resonance (CMR) is a versatile technique to non-invasively assess cardiovascular hemodynamics. With developing technology, choice in sequences and acquisition parameters is expanding and it is important to assess if data acquired with these different variants can be directly compared, especially when combining datasets within research studies. For example, sequences may allow a choice in gating techniques or be limited to one method, yet there is not a direct comparison investigating how gating selection impacts quantifications of the great vessels, semilunar and atrioventricular valves and ventricles. Thus, this study investigated if quantifications across the heart from contemporary 4D flow sequences are comparable between two commonly used 4D flow sequences reliant on different ECG gating techniques.

**Methods:**

Forty participants (33 healthy controls, seven patients with coronary artery disease and abnormal diastolic function) were prospectively recruited into a single-centre observational study to undergo a 3T-CMR exam. Two acquisitions, a k-t GRAPPA 4D flow with prospective gating (4D_prosp_) and a modern compressed sensing 4D flow with retrospective gating (4D_retro_), were acquired in each participant. Images were analyzed for volumes, flow rates and velocities in the vessels and four valves, and for biventricular kinetic energy and flow components. Data was compared for group differences with paired *t*-tests and for agreement with Bland-Altman and intraclass correlation (ICC).

**Results:**

Measurements primarily occurring during systole of the great vessels, semilunar valves and both left and right ventricles did not differ between acquisition types (*p* > 0.05 from *t*-test) and yielded good to excellent agreement (ICC: 0.75–0.99). Similar findings were observed for the majority of parameters dependent on early diastole. However, measurements occurring in late diastole or those reliant on the entire-cardiac cycle such as flow component volumes along with diastolic kinetic energy values were not similar between 4D_prosp_ and 4D_retro_ acquisitions resulting in poor agreement (ICC < 0.50).

**Discussion:**

Direct comparison of measurements between two different 4D flow acquisitions reliant on different gating methods demonstrated systolic and early diastolic markers across the heart should be compatible when comparing these two 4D flow sequences. On the other hand, late diastolic and intraventricular parameters should be compared with caution.

## Introduction

Interest in whole heart 4D flow cardiovascular magnetic resonance imaging (CMR) is rapidly growing as there are numerous parameters that can be derived from just a single acquisition including flow, pulse wave velocity, wall shear stress, pressure loss, energy loss, kinetic energy (KE) and particle tracking. Early use of cardiovascular 4D flow imaging was focused on aortic applications (1, 2). Quantification of transvalvular and intraventricular hemodynamics has gained traction over the last few years, as deeper understanding of left, and right heart flow patterns can increase the knowledge about the pathophysiology of cardiovascular diseases. Advancements in dynamic valve tracking accounting for the rapid and angular movement of the annuli allows for the quantification of valve function and regurgitation (3, 4). Importantly, flow through the atrioventricular valves and intraventricular analysis provides insight into diastolic function (5–9). Already in the 1990s the potential of intraventricular flow was published (10, 11) but the release of analysis software by multiple vendors in recent years has allowed more sites to utilize this technique and publish findings on whole-heart 4D flow (12–15). The rise in 4D flow applications is also driven by newer and faster 4D flow sequences which can be implemented easier into examinations. As a result, there are a variety of sequences, and parameters in use. As the choice of techniques increases with development, validating how 4D acquisitions compare to each other is necessary to assess if data acquired with these different variants can be directly compared, especially when comparing data between imaging sites or for combining datasets within research studies. Various groups have already shown reproducibility of 4D flow analysis when comparing longitudinal results, measurements between sites, different sequence types, magnetic field strengths, and acceleration factors (13, 16–20). In addition to different types of sequences with improving acceleration factors, image acquisition factors such as ECG gating may differ. 4D flow sequences may be limited to a specific ECG gating method or allow the imaging technician a choice of prospective or retrospective gating when acquiring data based on the individual patient. Consensus documents recommend to acquire images with retrospective gating, and if prospective gating is used to consider how data may be impacted by incomplete temporal coverage (21). Retrospective gating is ideal because it captures data for the entire cardiac cycle, however it is not available for all 4D sequences, and it is problematic in patients with varying RR intervals. Prospective gating helps overcome this limitation, but it can lead to the loss of the last percentages of the cardiac cycle, depending on the difference between the preselected acquisition window and the actual duration of the heart cycle when scanning. While both types of ECG gating have long been used (11, 22, 23), the option to choose, based on patient need may not be available in all sequence variants. For example, one of the conventional sequences assessed in this study is limited to only prospective gating, while one of the newer manufacture product sequences allows a choice for ECG gating. As these are two sequence types available for Siemens 3 T scanners, there was a need to investigate the compatibility of measurements prior to comparing 4D measurements from these sequence types in longitudinal and multicentre research studies.

The primary aim of this study was to compare in healthy controls the results from whole heart quantifications between two commonly used 4D flow sequences, a retrospective gated compressed sensing 4D flow sequence (4D_retro_) and a conventional 4D flow sequence with prospective gating (4D_prosp_). A secondary aim was then to highlight in a small sample of patients with abnormal diastolic function the agreement and discrepancies between measurements from the two 4D acquisitions.

## Methods

### Study and participant details

Participants were prospectively recruited into a single-centre observational study to undergo a CMR exam for research purposes. All participants (*n* = 40) provided informed written consent. The primary cohort consisted of 33 young healthy controls, defined as subjects aged 18–45 years and without cardiovascular or respiratory disease. To investigate the impact of different diastolic inflow patterns, a small subgroup of seven patients were included. Patients had coronary heart disease and known abnormal diastolic function, diagnosed in a clinical echocardiography exam. Patients were in sinus rhythm at the time of the exam. This study was approved and carried out in accordance with the Cantonal Research Ethics Board Bern, Switzerland (#2020-01258) and complies with the declaration of Helsinki.

### Imaging protocol

Participants underwent a single CMR visit (3.0 Tesla scanner, MAGNETOM Vida or Prisma, Siemens Healthineers, Germany). Standard cine sequences were acquired in short-axis and long-axis planes of the heart for volumetric assessment (typical parameters: retrospective-gated, 30 phases, TR/TE: 3.1 ms/1.38 ms, flip angle 45°, bandwidth 962 Hz/Px, spatial resolution 1.9 × 1.9 × 8.0 mm^3^). Two 4D acquisitions were then acquired in each participant in random order and parameters were adjusted to be as similar as possible between the two variations (Table 1). In all healthy controls and one patient, both sequences were acquired without contrast agent. In the remaining patients, both 4D flow sequences were acquired at least 10 min or later after administration of a gadolinium-based contrast agent.

**Table 1:** Acquisition parameters.

	4D_prosp_	4D_retro_
Breathing pattern	Free-breathing	Free-breathing
VENC	120 cm/s	120 cm/s
Temporal resolution	40.8 ms	37–42 ms
TR/TE	5.1 ms/2.68 ms	5.2 ms/2.47 ms
Flip angle	7°	8°
Bandwidth	450 Hz/Px	460 Hz/Px
Acquired voxel size	2.25 × 3.08 × 3.00 mm^3^	2.25 × 2.75 × 2.50 mm^3^
Reconstructed Voxel size	2.25 × 2.25 × 3.00 mm^3^	2.25 × 2.25 × 2.50 mm^3^
Slices per slab	52–64	56–80
Acquisition time—actual^a^	636 ± 154 s	396 ± 127 s
Acceleration factor	5.0	7.6
Arrhythmia rejection	No	No

^a^ Actual acquisition time is mean ± SD of all participants acquired from the DICOM tags of each acquired dataset. VENC, velocity encoding; TE, repetition time; TE, echo time; Hz, hertz; Px, pixel.

One sequence (4D_prosp_) was the sequence traditionally used at our institution using a k*-*t generalized autocalibrating partially parallel acquisition (GRAPPA, acceleration factor, undersampling R = 5, R_net _= 4.3) that only has capabilities for prospective gating. The other 4D flow sequence was a contemporary compressed sensing (*R* = 7.6) product sequence which provides the option for retrospective gating (4D_retro_). For both acquisitions, a transthoracic block was placed on a sagittal orientation ensuring full coverage of the heart and thoracic aorta including the top of the aortic arch and the descending aorta to at least the diaphragm. Both acquisitions were acquired during free-breathing using a diaphragmatic respiratory navigator. Both sequences were single-VENC, which was set at 120 cm/s and adjusted if required. For the ECG-prospectively triggered sequence (4D_prosp_), the acquisition window was defined by the technician to fit within the RR-interval, acquiring as much of the cycle as possible with time reserved for acquisition of the navigator (temporal resolution: 40.8 ms, TR/TE: 5.1 ms/2.68 ms, flip angle 7°, bandwidth 450 Hz/Px, acquired voxel size was 2.25 × 3.08 × 3.00 mm^3^ reconstructed to 2.25 × 2.25 × 3.00 mm^3^) (23, 24). The second 4D acquisition was retrospectively gated (4D_retro_), and phase number was defined prior to the acquisition based on the RR-interval (targeted temporal resolution: 37–42 ms, TR/TE: 5.2 ms/2.47 ms, flip angle 8°, bandwidth 460 Hz/Px, acquired spatial resolution of 2.25 × 2.75 × 2.50 mm^3^ reconstructed to 2.25 × 2.25 × 2.50 mm^3^). For the 4D_retro_ sequence, the kz-ky sampling pattern uses a variable density spiral phyllotaxis pattern for the compressed sensing while minimizing respiration induced artifacts by acquiring central k-space data during end-expiration and outer k-space during inspiration (25).

### CMR image analysis

Images were recoded and analysed by blinded readers. Standard volumetric assessment of the LV and RV were acquired from the cines, following placement of endocardial and epicardial contours on end-diastolic and end-systolic frames. Whole heart 4D flow was then quantified.

### Vascular analysis

**Figure 1:**
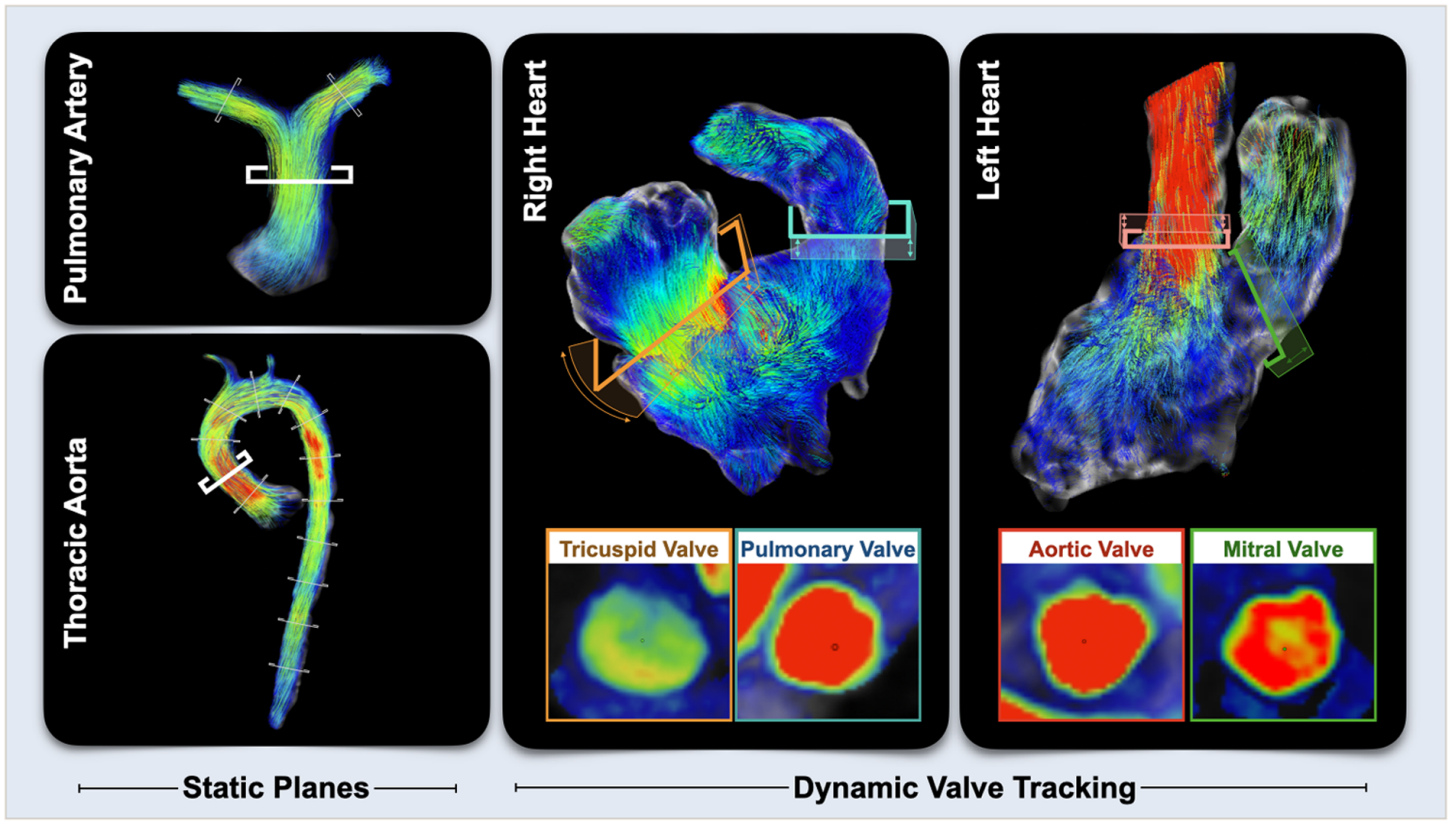
4D flow quantification. For vascular assessments, multiple static planes were placed on the pulmonary artery and aorta with a focus on the main pulmonary artery and ascending aorta. For valvular and ventricular analyses, planes were place on the annulus of the four valves and adjusted throughout the cardiac cycle tracking the movement of valve.

A centreline was placed in the aorta from the aortic sinus to the descending aorta at the level of the diaphragm (Figure 1). A static plane was placed on the mid-ascending aorta at the level of the pulmonary artery following the standard measurement locations defined in the 2022 ACC/AHA guidelines (26). The lumen was then contoured throughout the cardiac cycle. For the calculation of pulse wave velocities, 11 equally spaced additional planes were then added onto the ascending and descending aorta until the level of the diaphragm, taking advantage of the full volumetric coverage of the thoracic aorta (27, 28). Flow was also quantified in the main pulmonary artery. From the vascular analysis, total volumes, peak flow rate, velocity and pressure gradients were calculated.

### Valvular and intraventricular analysis

**Figure 2:**
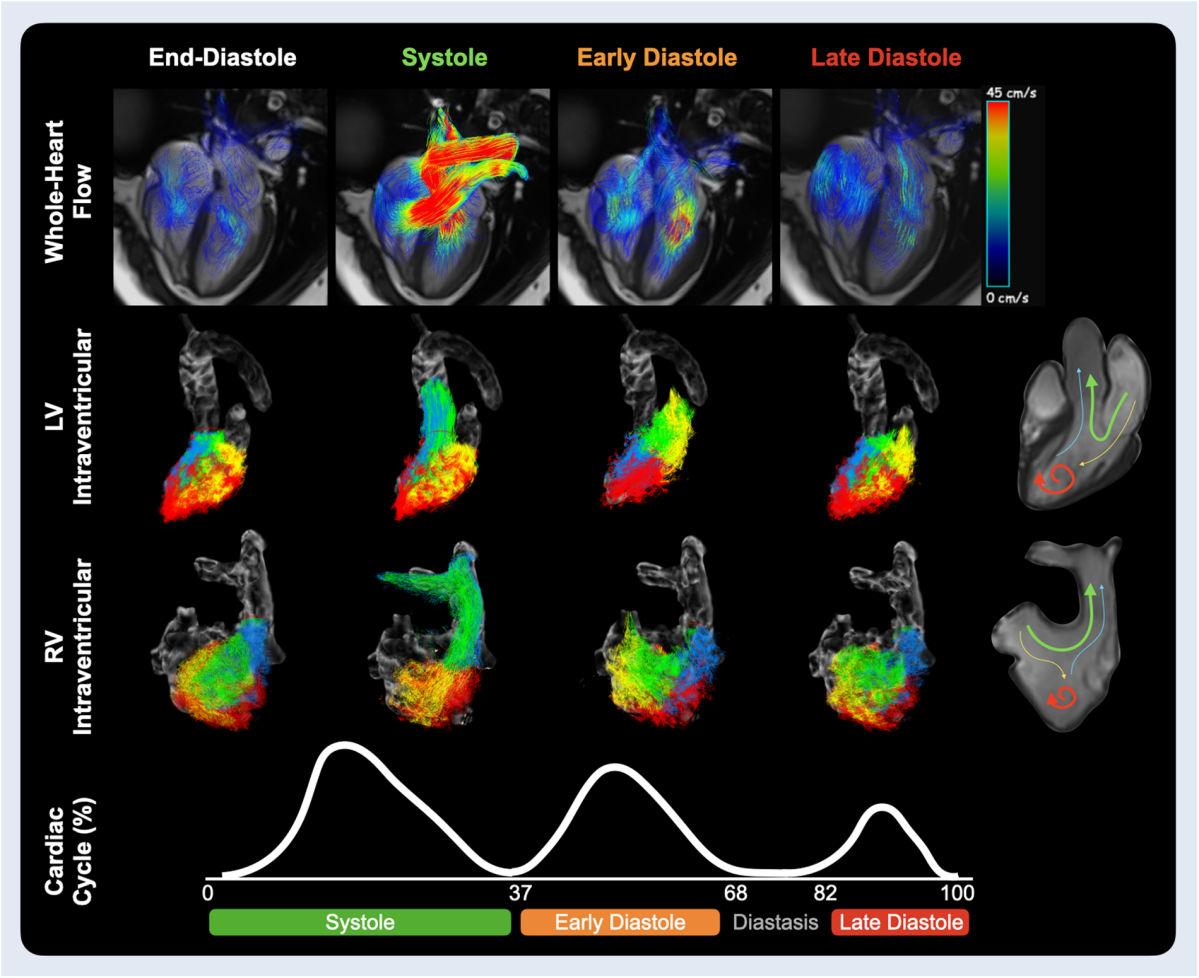
Ventricular hemodynamics. Velocity path lines through the heart are shown on the top row for the end-diastolic phase, mid systole and at the peak inflow of early and late diastole. The middle rows show the ventricular components for the left and right heart respectively. These flow components include direct flow (green), blood that enters and leaves the ventricle during the same heartbeat; retained inflow (yellow), blood that enters the ventricle but is only ejected during the next heartbeat; delayed ejection (blue), blood that is being ejected but entered from the previous heartbeat; and residual volume (red), blood that stays for at least two or more heartbeats within the ventricle. The sum of all four components provides global intraventricular measurements. Timing of systolic, early and late diastolic waves are acquired from the average wave duration of aortic and mitral flow analysis of 32 healthy controls (Supplementary Results).

Using a dynamic valve tracking module, the aortic and mitral valve were contoured at the level of the annulus throughout the cardiac cycle. For the right heart, contours were placed on the pulmonary and tricuspid valves. Transvalvular flows were quantified, and peak flow rates and velocities were quantified for the semilunar valves (aortic and pulmonary) and for the early and late diastolic (atrial contribution) inflow of the atrioventricular valves (mitral and tricuspid). On the 4D_retro_ datasets, the time of the start and end of each flow wave was recorded and expressed in both ms and as a %-of the cardiac cycle (Figure 2).

Intraventricular measures were quantified by the software based on the valvular contours and selection of an isometric relaxation phase. Peak systolic, early and late diastolic KE was calculated for each ventricle. Additionally, the ventricle was assessed for functional components focusing on direct flow, blood that enters and is ejected from the ventricle within a single cardiac cycle, and residual volume, blood that stayed in the ventricle for the same time frame (Figure 2). All analysis was performed with cvi^42^ (version 5.17, Circle Cardiovascular Imaging, Calgary, Canada).

### Data and statistical analysis

All 4D measures of the great vessels, valves and ventricles were compared first with a paired *t*-test. In the healthy controls, agreement was compared using a Bland-Altman test and a two-way mixed intraclass correlation (ICC) coefficient for absolute agreement. ICC coefficients >0.90 indicated excellent agreement, 0.75–0.90 good agreement, 0.50–0.75 moderate agreement, and poor agreement is represented by values <0.50. For both the 4D_prosp_ and 4D_retro_, the difference in transvalvular flow volumes (ml) between the semilunar valves and the atrioventricular valves were quantified to assess if all diastolic inflow was accounted for in comparison to systolic outflow. The EDV from the RV and LV of both 4D sequences were then compared to the EDV acquired from standard volumetric assessment to assess if all ventricular volume was detected by the 4D techniques. Statistical significance was defined with a two-sided *p*-value of <0.05. GraphPad Prism version 10 (GraphPad Software, La Jolla, California, USA), and IBM SPSS Statistics 26 (IBM, Armonk, NY, USA) were used for statistical analysis.

## Results

### Healthy participant characteristics

**Figure 3:**
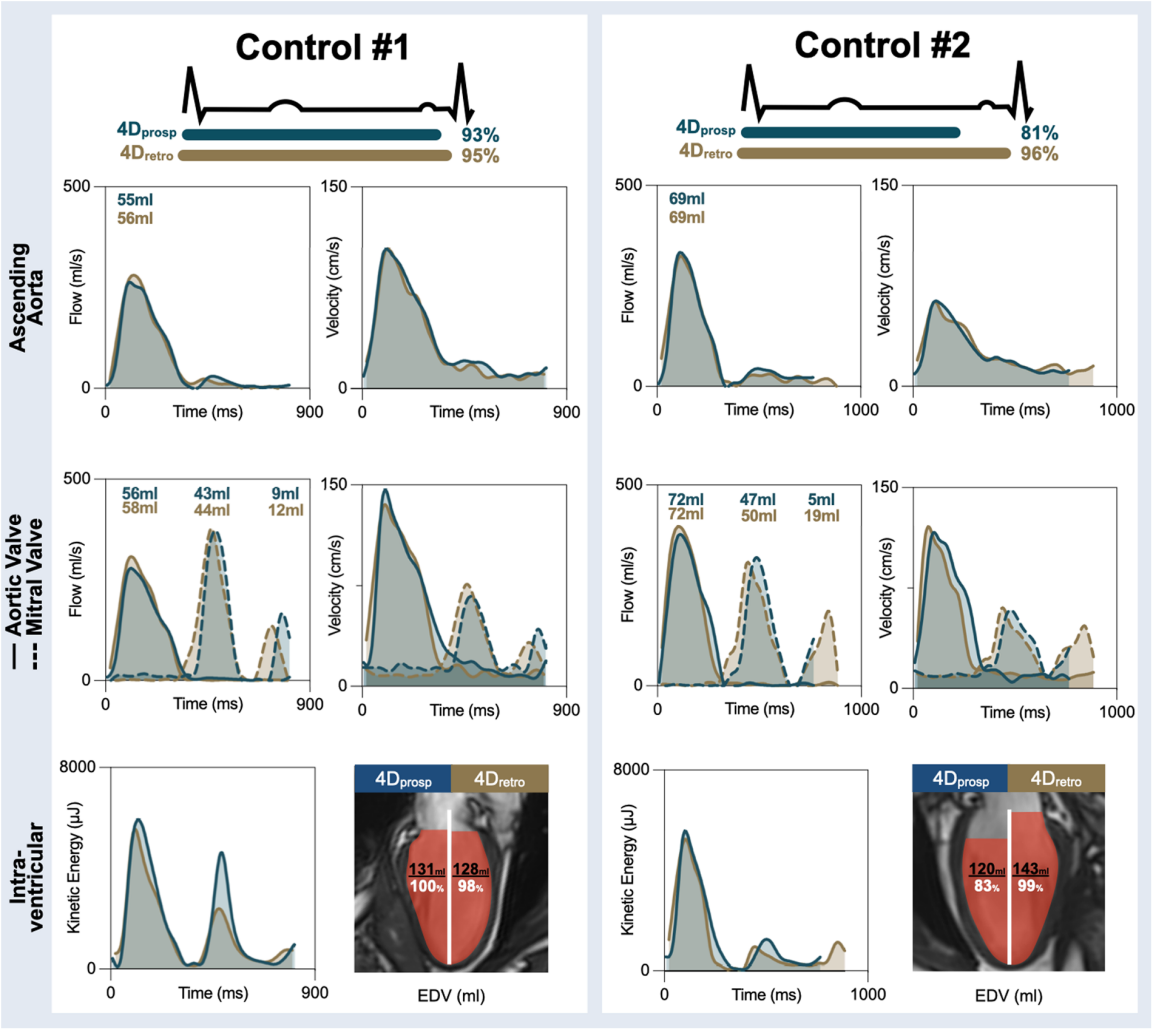
Healthy control examples. Measurements across the cardiac cycle are shown for flow and velocity in the ascending aorta (top), through the aortic valve (solid line) and mitral valve (dotted line) for both the prospectively gated (4D_prosp_, blue) and retrospectively gated (4D_retro_, gold) 4D flow. Total effective volume per wave is shown for the flow curves. The bottom row depicts global kinetic energy and the total end-diastolic volume (EDV) detected by 4D flow in comparison to EDV measured by standard volume analysis from cine images. In both controls, measures during the systolic phase of the cardiac cycle depict a significant overlap. In control #1, 93% of the cardiac cycle was acquired with the 4D_prosp_ and the A-wave can be observed with transmitral flow and velocities and intraventricular kinetic energy resulting in similar EDV. In control #2, only 81% of the cycle was obtained and a significant portion of the A-wave was lost. As a result, EDV was underestimated by the 4D_prosp_.

Thirty-two healthy controls were included into the analysis as one control was excluded due to a heartrate difference of 31 bpm between 4D acquisitions. Detailed patient characteristics can be found in Supplementary Table 1. From the included 32 controls, 86 ± 6% of the RR interval was acquired with the 4D_prosp_ sequence. The coverage of the RR interval ranged from 69% to 95% (Figure 3). This was significantly less than the 96 ± 1% of the RR interval acquired with the 4D_retro_ sequence (*p* < 0.01, range: 93%–98%). Mean acquisition time was longer with the 4D_prosp_ (645 ± 148 s) than the 4D_retro_ (389 ± 139 s, *p* < 0.01). Heart rate did not significantly differ between acquisitions (62 ± 11 bpm vs. 63 ± 11 bpm, *p* = 0.30), neither did the estimated navigator efficiency (68% [IQR: 61–74] vs. 77% [IQR: 65–82], *p* = 0.09).

### Comparisons of the great vessels in healthy controls

As a measure of the entire thoracic aorta, pulse wave velocity was similar between the 4D_prosp_ and 4D_retro_ (5.0 ± 0.8 m/s vs. 5.0 ± 0.8 m/s, *p* = 0.72) in the healthy controls. Correspondingly, a good ICC was observed for this parameter (ICC = 0.86, *p* < 0.01). Quantification through a static plane on the ascending aorta (Table 2) similarly showed the same volumes, peak flows and velocities with both sequences with good to excellent ICC (all >0.87, *p* < 0.01). Similar ICC findings were observed with measurements of the main pulmonary artery, although minutely higher volumes (75 ± 15 ml vs. 78 ± 14 ml, *p* = 0.02) and flow rates (314 ± 86 ml/s vs. 329 ± 78 ml/s, *p* = 0.04) were detected with the 4D_retro_, while peak velocities were similar (80 ± 14 cm/s vs. 80 ± 12 cm/s, *p* = 0.70).

**Table 2:** 4D measures of the great vessels of healthy controls.

	4D_prosp_	4D_retro_	Bias (%)	*p*	ICC	*p*
%-Cardiac cycle acquired (range)	85 ± 6 [69–95]	96 ± 1 [93–99]	−11 ± 6	<0.01	–	–
Aorta						
Thoracic aorta						
Pulse wave velocity (m/s)	5.0 ± 0.8	5.0 ± 0.8	1 ± 11	0.72	0.86	<0.01
Ascending aortic plane						
Total volume (ml)	73 ± 13	73 ± 11	0 ± 9	0.13	0.91	<0.01
Cardiac output (L/min)	4.5 ± 1.2	4.6 ± 1.1	−2 ± 12	0.10	0.93	<0.01
Max flow rate (ml/s)	333 ± 86	328 ± 64	1 ± 10	0.72	0.94	<0.01
Max velocity (cm/s)	98 ± 21	95 ± 16	4 ± 13	0.89	0.88	<0.01
Max pressure gradient (mmHg)	4.1 ± 1.8	3.7 ± 1.2	10 ± 27	0.69	0.87	<0.01
Main pulmonary artery						
Total volume (ml)	75 ± 15	78 ± 14	−4 ± 10	0.02	0.92	<0.01
Cardiac output (L/min)	4.7 ± 1.4	4.9 ± 1.3	−5 ± 12	0.04	0.92	<0.01
Max flow rate (ml/s)	314 ± 86	329 ± 78	−5 ± 11	0.02	0.93	<0.01
Max velocity (cm/s)	80 ± 14	80 ± 12	1 ± 12	0.701	0.81	<0.01
Max pressure gradient (mmHg)	2.6 ± 1.0	2.6 ± 0.7	4 ± 26	0.60	0.78	<0.01

Mean ± SD are shown for vascular measurements of healthy controls (*n* = 32) from both 4D sequences which were prospectively (4D_prosp_) and retrospectively gated (4D_retro_). A percent-bias is reported using the 4D_retro_ sequence as reference. ICC, intraclass correlation.

### Comparisons of transvalvular measurements in healthy controls

With the 4D_retro_ sequence, the complete transvalvular flow could be quantified in all participants for the systolic, (aortic and pulmonary valve) early and late (mitral and tricuspid valve) diastolic phases of the cardiac cycle (Figure 3). With the 4D_prosp_, the entire aortic and pulmonary valve flow was similarly detected in all participants, while the entire early flow through the mitral and tricuspid valves were detected in 31/32 controls, with the E-wave truncated in a control where only 69% of the cardiac cycle was acquired. However, for late diastolic flow, the start of the A-wave was only detected in 22/32 controls, with the entire A-wave visualized in just 2/32 controls who had 93% and 95% of the cycle captured (detailed further in Supplementary Text).

For both the aortic valve and the pulmonary valve, there were no differences between the 4D_prosp_ and 4D_retro_ measurements (Table 3). Total volume through the aortic valve (74 ± 12 ml vs. 77 ± 14 ml, *p* = 0.05) and pulmonary valve (76 ± 14 ml vs. 76 ± 14 ml, *p* = 0.96) was comparable as were the peak velocities (aortic valve: 115 ± 12 cm/s vs. 116 ± 12 cm/s, *p* = 0.13, pulmonary valve: 82 ± 13 cm/s vs. 83 ± 15 cm/s, *p* = 0.59). As a result, agreement from ICC analysis ranged from good to excellent (Table 3). Discrepancy occurred when comparing 4D measures through the atrioventricular valves during diastole. 4D_prosp_ underestimated total volume through the mitral valve by 21% in comparison to the 4D_retro_ (*p* < 0.01). This was primarily due to the loss of volume detected in late diastole as the 4D_prosp_ quantified only 3 ± 3 ml from late diastolic A-wave while the 4D_retro_ quantified 15 ± 5 ml. On the other hand, there was only minimal difference in early diastolic volume (56 ± 11 ml vs. 60 ± 12 ml, *p* < 0.01). In fact, the early diastolic measures yielded good agreement (all ICC > 0.75), with peak velocity of the E-wave appearing to be one of the most consistent measures for the mitral valve (74 ± 13 cm/s vs. 75 ± 17 cm/s, *p* = 0.64). As a result of incomplete interrogation of late diastolic flow with the 4D_prosp_ acquisition, the peak flow rate and velocity of the A-wave were not reported.

**Table 3:** 4D valvular measurements of healthy controls.

	4D_prosp_	4D_retro_	%-bias	*p*	ICC	*p*
Aortic valve						
Total volume (ml)	74 ± 12	77 ± 14	3 ± 9	0.05	0.89	<0.01
Cardiac output (L/min)	4.6 ± 1.1	4.8 ± 1.1	4 ± 11	0.06	0.91	<0.01
Peak pressure gradient (mmHg)	5.2 ± 1.1	5.5 ± 1.1	2 ± 12	0.11	0.91	<0.01
Peak flow rate (ml/s)	369 ± 75	378 ± 76	2 ± 9	0.18	0.94	<0.01
Peak velocity (cm/s)	115 ± 12	116 ± 12	1 ± 6	0.13	0.91	<0.01
Mitral valve						
Total volume (ml)	60 ± 12	77 ± 13	−21 ± 12	<0.01	0.51	<0.01
Aortic-mitral valve volume mismatch (Δml, [%])	14 ± 9 [24 ± 18]	0 ± 4 [0 ± 5]	–	<0.01	–	–
Max pressure gradient (mmHg)	2.3 ± 0.8	2.3 ± 0.9	9 ± 40	0.96	0.83	<0.01
Early diastolic (E)						
E-volume (ml)	56 ± 11	60 ± 12	−6 ± 10	0.02	0.87	<0.01
E-peak flow rate (ml/s)	406 ± 74	374 ± 77	11 ± 21	0.05	0.78	<0.01
E-peak velocity (cm/s)	74 ± 13	75 ± 17	1 ± 15	0.64	0.86	<0.01
Late diastolic (A)						
A-volume (ml)	3 ± 3	15 ± 5	−78 ± 24	<0.01	0.03	0.77
A-peak flow rate (ml/s)	–	140 ± 46	–	–	–	–
A-peak velocity (cm/s)	–	35 ± 8	–	–	–	–
E/A- peak flow rate	–	2.9 ± 1.1	–	–	–	–
E/A- peak velocity	–	2.3 ± 0.8	–	–	–	–
Pulmonary valve						
Total volume (ml)	76 ± 14	76 ± 14	0 ± 5	0.96	0.99	<0.01
Cardiac output (L/min)	4.7 ± 1.3	4.7 ± 1.3	1 ± 9	0.46	0.80	<0.01
Peak pressure gradient (mmHg)	2.8 ± 0.9	2.8 ± 1.1	2 ± 7	0.58	0.77	<0.01
Peak flow rate (ml/s)	350 ± 79	357 ± 80	2 ± 7	0.15	0.97	<0.01
Peak velocity (cm/s)	82 ± 13	83 ± 15	1 ± 10	0.59	0.83	<0.01
Tricuspid valve						
Total volume (ml)	58 ± 12	71 ± 13	−17 ± 12	<0.01	0.60	<0.01
Pulmonary-tricuspid valve volume mismatch (Δml, [%])	17 ± 10 [22 ± 11]	5 ± 6 [6 ± 7]	–	<0.01	–	–
Max pressure gradient (mmHg)	1.2 ± 1.1	1.2 ± 0.5	13 ± 19	0.48	0.67	0.01
Early diastolic (E)						
E-volume (ml)	55 ± 11	56 ± 11	0 ± 13	0.66	0.86	<0.01
E-peak flow rate (ml/s)	324 ± 64	292 ± 71	14 ± 19	<0.01	0.82	<0.01
E-peak velocity (cm/s)	54 ± 7	53 ± 9	3 ± 13	0.52	0.79	<0.01
Late diastolic (A)						
A-volume (ml)	2 ± 3	14 ± 7	−77 ± 25	<0.01	0.07	0.26
A-peak flow rate (ml/s)	–	129 ± 52	–	–	–	–
A-peak velocity (cm/s)	–	28 ± 9	–	–	–	–
E/A- peak flow rate	–	2.6 ± 1.2	–	–	–	–
E/A- peak velocity	–	2.0 ± 0.7	–	–	–	–

Mean ± SD are shown for transvalvular measurements of healthy controls (*n* = 32) from both 4D sequences which were prospectively (4D_prosp_) and retrospectively gated (4D_retro_). A percent-bias is reported using the 4D_retro_ sequence as reference. A, late diastole (atrial contribution); E, early diastole; ICC, intraclass correlation.

The same trends were observed for the tricuspid valve where the 4D_prosp_ underestimated total volume by 17% in comparison to the 4D_retro_. While early diastolic flow was similar (55 ± 11 ml vs. 56 ± 11 ml, *p* = 0.66), late diastolic volume was missing with the 4D_prosp_ (2 ± 3 ml vs. 14 ± 7 ml, *p* < 0.01). As in the mitral valve, peak velocity of the E-wave was the same for both sequences (54 ± 7 cm/s vs. 53 ± 9 cm/s, *p* = 0.52), with good agreement for the early diastolic measures (all ICC >0.80).

### Comparisons of intraventricular measures in healthy controls

In comparison to EDV, acquired from the standard quantification of 2D cines, the 4D_prosp_ significantly underestimated the LV EDV by 28 ± 29 ml (*p* < 0.01), while the 4D_retro_ showed no mismatch (−1 ± 13 ml, *p* = 0.94). Similarly, the 4D_prosp_ underestimated RV EDV by 37 ± 33 ml (*p* < 0.01), while the 4D_retro_ did not (2 ± 17 ml, *p* = 0.29). Volumes of the intraventricular components, direct flow and residual volume, were generally underestimated by the 4D_prosp_ for both ventricles in comparison to 4D_retro_ (Table 4). As a result, there was poor to no agreement (all ICC < 0.50) for the comparison of both volumes (ml) and percentage of the components.

**Table 4:** 4D intraventricular measurements of healthy controls.

	4D_prosp_	4D_retro_	%-Bias	*p*	ICC	*p*
Left ventricle						
End-diastolic volume (ml)	140 ± 23	167 ± 29	−15 ± 13	<0.01	0.47	<0.01
Direct flow (ml)	41 ± 9	47 ± 11	−9 ± 24	<0.01	0.47	0.02
Direct flow (%)	30 ± 7	28 ± 5	9 ± 28	0.22	0.27	0.19
Residual volume (ml)	36 ± 9	53 ± 17	−25 ± 41	<0.01	−0.10	0.67
Residual volume (%)	25 ± 6	32 ± 6	−16 ± 34	<0.01	−0.28	0.85
Systolic peak kinetic energy						
Total (µJ)	6,085 ± 2,326	6,146 ± 2,008	−1 ± 25	0.95	0.83	<0.01
– index_EDV_ (µJ/ml)	44 ± 15	37 ± 9	20 ± 30	0.01	0.70	<0.01
Direct flow (µJ)	3,750 ± 1,705	4,117 ± 1,599	−6 ± 29	0.15	0.77	<0.01
– index_DF_ (µJ/ml)	89 ± 26	87 ± 24	6 ± 32	0.74	0.73	<0.01
Residual volume (µJ)	246 ± 106	335 ± 185	−13 ± 53	0.03	0.48	0.02
– index_Res_ (µJ/ml)	7 ± 3	6 ± 2		0.33	0.77	<0.01
Early (E) diastolic peak kinetic energy						
Total (µJ)	4,447 ± 1,789	2,821 ± 1,318	81 ± 98	<0.01	0.36	0.03
– index_EDV_ (µJ/ml)	31 ± 10	17 ± 6	109 ± 101	<0.01	0.22	0.05
Direct flow (µJ)	2,287 ± 989	1,259 ± 692	95 ± 106	<0.01	0.23	0.11
– index_DF_ (µJ/ml)	55 ± 22	27 ± 13	18 ± 33	<0.01	0.01	0.26
Residual volume (µJ)	427 ± 206	436 ± 277	29 ± 87	0.98	0.11	0.38
– index_Res_ (µJ/ml)	12 ± 4	8 ± 3	66 ± 52	<0.01	0.45	<0.01
Late (A) diastolic peak kinetic energy						
Total (µJ)	–	1,068 ± 638	–	–	–	–
– index_EDV_ (µJ/ml)	–	6 ± 3	–	–	–	–
Direct flow (µJ)	–	407 ± 222	–	–	–	–
– index_DF_ (µJ/ml)	–	9 ± 4	–	–	–	–
Residual volume (µJ)	–	196 ± 140	–	–	–	–
– index_Res_ (µJ/ml)	–	4 ± 2	–	–	–	–
Right ventricle						
End-diastolic volume (ml)	163 ± 30	194 ± 35	−15 ± 11	<0.01	0.65	<0.01
Direct flow (ml)	43 ± 12	51 ± 12	−13 ± 30	<0.01	0.39	0.05
Direct flow (%)	26 ± 6	27 ± 7	−3 ± 35	0.73	0.20	0.27
Residual volume (ml)	43 ± 12	58 ± 17	−21 ± 27	0.10	0.38	0.02
Residual volume (%)	26 ± 5	30 ± 7	−7 ± 30	0.02	0.28	0.15
Systolic peak kinetic energy						
Total (µJ)	5,516 ± 1,907	6,067 ± 2,393	−3 ± 28	0.17	0.79	<0.01
– index_EDV_ (µJ/ml)	34 ± 10	31 ± 9	14 ± 30	0.02	0.78	<0.01
Direct flow (µJ)	3,507 ± 1,534	4,056 ± 1,654	−7 ± 37	0.04	0.82	<0.01
– index_DF_ (µJ/ml)	80 ± 20	77 ± 28	11 ± 31	0.23	0.79	<0.01
Residual volume (µJ)	272 ± 108	366 ± 184	−11 ± 46	0.03	0.21	0.23
– index_Res_ (µJ/ml)	6 ± 2	6 ± 2	11 ± 43	0.75	0.32	0.16
Early (E) diastolic peak kinetic energy						
Total (µJ)	2,384 ± 868	1,617 ± 593	54 ± 48	<0.01	0.58	<0.01
– index_EDV_ (µJ/ml)	15 ± 4	8 ± 3	82 ± 55	<0.01	0.34	0.01
Direct flow (µJ)	1,099 ± 517	673 ± 290	81 ± 94	<0.01	0.44	<0.01
– index_DF_ (µJ/ml)	25 ± 8	13 ± 5	115 ± 91	<0.01	0.26	0.01
Residual volume (µJ)	241 ± 98	260 ± 111	2 ± 45	0.54	0.46	0.05
– index_Res_ (µJ/ml)	6 ± 1	5 ± 1	29 ± 38	<0.01	0.40	0.03
Late (A) diastolic peak kinetic energy						
Total (µJ)	–	920 ± 490	–	–	–	–
– index_EDV_ (µJ/ml)	–	5 ± 2	–	–	–	–
Direct flow (µJ)	–	334 ± 212	–	–	–	–
– index_DF_ (µJ/ml)	–	6 ± 3	–	–	–	–
Residual volume (µJ)	–	153 ± 83	–	–	–	–
– index_Res_ (µJ/ml)	–	3 ± 1	–	–	–	–

Mean ± SD are shown for ventricular measurements of healthy controls (*n* = 32) from both 4D sequences, which were prospectively (4D_prosp_) and retrospectively gated (4D_retro_). Measures of agreement are displayed on the right. Global kinetic energies are indexed to end-diastolic volume (EDV) obtained from 4D analysis, while direct flow (DF) and residual volume (Res) are indexed to the respective component volume. A percent-bias is reported using the 4D_retro_ sequence as reference.

A, late diastole (atrial contribution); E, early diastole; KE, kinetic energy; ICC, intraclass correlation.

For intraventricular KE, good agreement was observed for global peak systolic KE of both LV (ICC = 0.83, *p* < 0.01) and RV (ICC = 0.79, *p* < 0.01) with no difference observed between the 4D_prosp_ and 4D_retro_ sequences (LV: 6,085 ± 2,326 µJ vs. 6,146 ± 2,008 µJ, *p* = 0.95, RV: 5,515 ± 1,907 µJ vs. 6,067 ± 2,393 µJ, *p* = 0.17). For the early diastolic peak KE, agreement worsened, and in both ventricles a significantly higher peak KE was observed with the 4D_prosp_ sequence (Table 4). Agreement was not assessed for late diastole, as peak A-wave could only be consistently observed with the 4D_retro_. Good agreement was also observed for the peak systolic KE of the direct flow component for the LV (3,750 ± 1,705 µJ vs. 4,117 ± 1,599 µJ *p* = 0.15, ICC = 0.77, *p* < 0.01) and for the RV (ICC = 0.82, *p* < 0.01), although a significant difference was still observed in the RV (3,507 ± 1,534 µJ vs. 4,056 ± 1,654 µJ, *p* = 0.04). Similar to global KE, agreement worsened for early diastolic KE, while agreement was poor for KE of residual volume.

### Patient cohort

**Figure 4:**
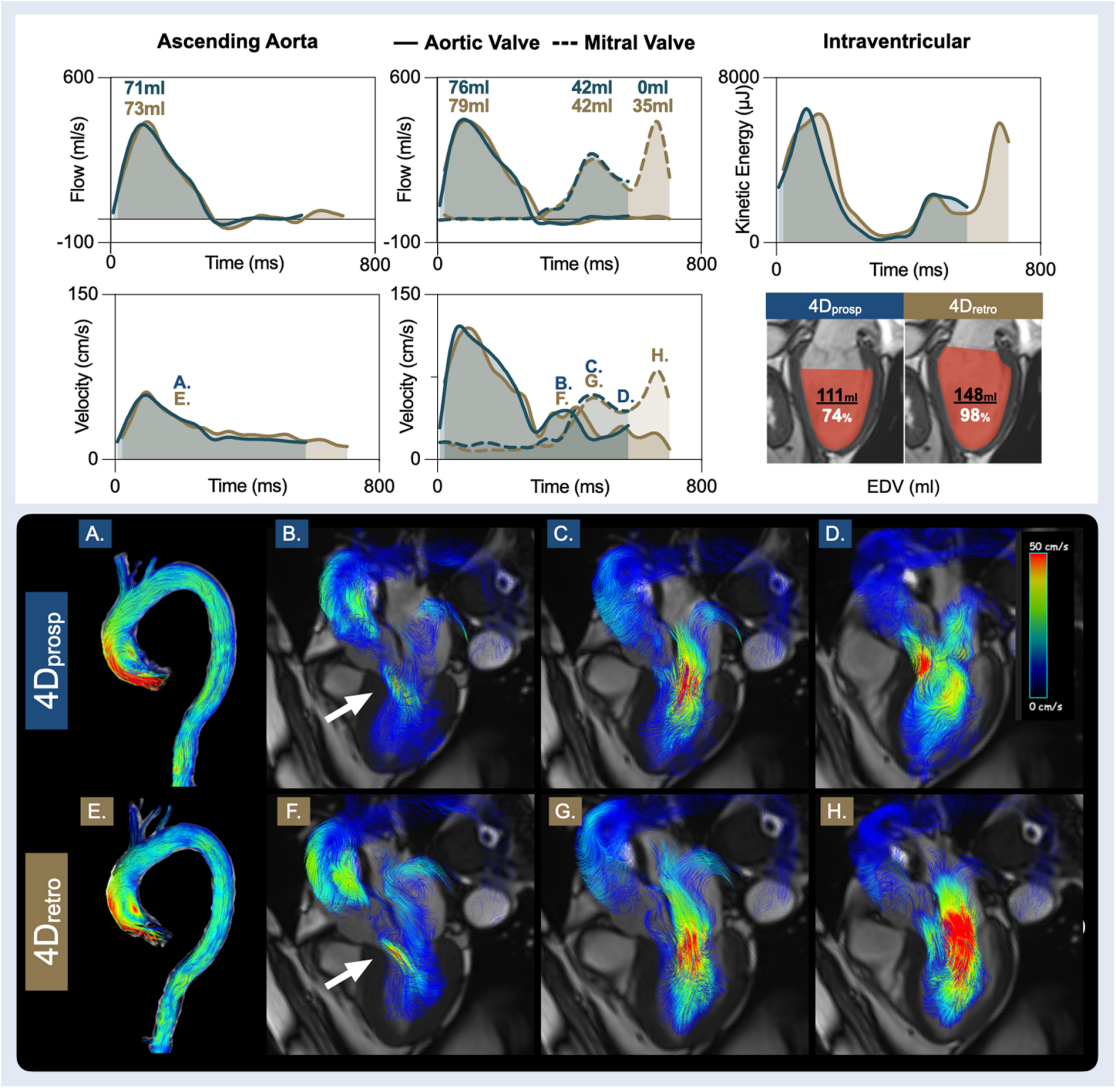
Patient with diastolic dysfunction. Presented are measurements from the prospective gated (4D_prosp_, blue, 80% cardiac cycle), and retrospective gated (4D_retro_, gold, 95% cardiac cycle) 4D flow of a 74-year-old male, with hypertension, known CAD and recent abdominal aortic repair surgery. A clinical echocardiography report diagnosed diastolic dysfunction grade I, light aortic valve regurgitation with an asymptomatic ascending aortic dilation (40 mm) and LV hypertrophy. The bottom two rows display 4D flow streamlines colour coded by velocity. The time point of each image (**A**–**F**) is labelled on the velocity graphs. It can be observed that despite an abnormal aorta (**A**/**E**), ascending aortic and aortic valve volumes, flows and velocities are similar during systole, and both depict the aortic valve regurgitation (**B**/**F**) occurring during diastole. Both sequences yielded similar findings for early diastole. Yet, due to the diastolic dysfunction, a significant portion of diastolic filling occurs in the final 150 ms that was not captured by prospective gating. As a result, it can be observed in the final phase captured by 4D_prosp_ (**D**), the ventricle is still filling. Consequently, neither true peak A-flow rate nor A-velocity was quantified. In image H with 4D_retro_, a higher A-flow rate and A-velocity was present in late diastole than early diastole (**G**), therefore, total diastolic transmitral volume, flow and end-diastolic volume (EDV) are underestimated.

Data was included from six patients (Supplementary Table S1). One patient was excluded from the analysis due to poor image quality of the 4D_retro_ sequence. The poor image quality of the excluded dataset appeared to be due to poor triggering, since two partial systolic flow waves were observed in the aorta at different timepoints of the cardiac cycle. With the 4D_prosp_, 81 ± 4% (range: 76%–87%) of the cardiac cycle was acquired, in comparison to 95 ± 1% (range: 95%–96%) acquired by the 4D_retro_ (*p* < 0.01), and acquisition time was 590 ± 187 s and 402 ± 66 s (*p* = 0.04), respectively. Estimated navigator efficiency did not significantly differ (72% [IQR: 63–83] vs. 77% [IQR: 70–87], *p* = 0.69). Similar to the healthy controls, the analysis of the six patients demonstrated that there was no difference in the majority of systolic measures (Tables 5, 6), including matching pulse wave velocities, flows, volumes and velocities through the aorta, aortic valve and the pulmonary artery and valve between the 4D_prosp_ and 4D_retro_ acquisition. Additionally, early diastolic vascular and valvular measurements were identical. Yet as in the healthy controls, incomplete acquisition of late diastole resulted in varying intraventricular analysis. As shown in the patient example of Figure 4, the measurements through the systolic and early diastolic phases of the cardiac cycle were consistent between the sequences. The displayed patient had echocardiographically defined diastolic dysfunction grade I. It can be observed that almost half of the trans-mitral flow (*E* = 42 ml, *A* = 35 ml) occurred during the A-wave in the final 150 ms of the cardiac cycle (21%). The A-wave showed a higher flow rate and velocity compared to the E-wave. As a result, the 4D_prosp_ sequence that captured 80% of the cardiac cycle in this patient did not capture late diastole. Consequently, total mitral volume and EDV were significantly underestimated. Moreover, while systolic KE was similar, the calculation of the diastolic energy profiles for left ventricular and flow components also differed.

**Table 5:** 4D measurements of the great vessels and valves in the patient cohort.

	Left heart			Right heart		
	4D_prosp_	4D_retro_	*p*	4D_prosp_	4D_retro_	*p*
Vessels	Ascending aorta			Pulmonary artery		
Total volume (ml)	73 ± 13	66 ± 12	0.08	72 ± 10	75 ± 9	0.38
Cardiac output (L/min)	4.9 ± 0.9	4.5 ± 1.1	0.23	4.9 ± 1.0	5.2 ± 0.8	0.36
Max flow rate (ml/s)	322 ± 66	287 ± 12	0.04	319 ± 56	330 ± 62	0.65
Max velocity (cm/s)	75 ± 24	70 ± 25	0.20	78 ± 5	81 ± 21	0.73
Max pressure gradient (mmHg)	2.5 ± 1.6	2.2 ± 1.4	0.25	2.4 ± 0.3	2.8 ± 1.6	0.63
Pulse wave velocity (m/s)	9.0 ± 3.8	8.4 ± 2.8	0.69			
Semilunar valves	Aortic valve			Pulmonary valve		
Total volume (ml)	71 ± 11	71 ± 11	0.97	72 ± 11	73 ± 9	0.75
Cardiac output (L/min)	4.8 ± 1.0	4.9 ± 1.2	0.70	4.9 ± 1.0	5.0 ± 1.0	0.55
Max pressure gradient (mmHg)	8.2 ± 3.9	6.9 ± 1.6	0.24	3.5 ± 1.4	3.4 ± 1.4	0.66
Max flow rate (ml/s)	342 ± 66	345 ± 71	0.88	364 ± 64	356 ± 71	0.52
Max velocity (cm/s)	142 ± 25	130 ± 16	0.22	92 ± 17	91 ± 17	0.68
Atrioventricular valves	Mitral valve			Tricuspid valve		
Total volume (ml)	51 ± 7	70 ± 11	0.03	45 ± 7	67 ± 12	0.03
Semilunar-atrioventricular volume mismatch (Δml, [%])	−20 ± 10 [−40 ± 20]	−2 ± 3 [−3 ± 4]	<0.01	−28 ± 14 [−37 ± 17]	−7 ± 8 [−9 ± 10]	0.03
Max pressure gradient (mmHg)	1.6 ± 0.7	1.6 ± 0.8	0.95	1.1 ± 0.3	1.2 ± 0.8	0.66
Early diastolic (E)						
E-volume (ml)	49 ± 8	45 ± 11	0.22	46 ± 10	42 ± 12	0.27
E-peak flow rate (ml/s)	345 ± 88	297 ± 77	0.34	258 ± 88	238 ± 56	0.61
E-peak velocity (cm/s)	63 ± 13	58 ± 16	0.73	44 ± 10	41 ± 8	0.42
Late diastolic (A)						
A-volume (ml)	2 ± 3	27 ± 14	<0.01	2 ± 3	27 ± 14	<0.01
A-peak flow rate (ml/s)	–	270 ± 83	–	–	270 ± 46	–
A-peak velocity (cm/s)	–	57 ± 11	–	–	49 ± 21	–
E/A- max flow rate	–	1.3 ± 0.5	–	–	0.9 ± 0.1	–
E/A- max velocity	–	1.2 ± 0.4	–	–	0.9 ± 0.2	–

Mean ± SD are shown for vascular and valvular measurements of patients with cardiovascular disease (*n* = 6) from both 4D sequences which were prospectively (4D_prosp_) and retrospectively gated (4D_retro_).

**Table 6:** 4D intraventricular measurements in the patient cohort.

	Left heart			Right heart		
	4D_prosp_	4D_retro_	*p*	4D_prosp_	4D_retro_	*p*
Intraventricular	Left ventricle			Right ventricle		
End-diastolic volume (ml)	133 ± 20	163 ± 26	<0.01	140 ± 34	186 ± 39	<0.01
Direct flow (ml)	37 ± 12	41 ± 14	0.40	34 ± 8	45 ± 15	0.08
Direct flow (%)	29 ± 10	26 ± 8	0.27	25 ± 6	24 ± 5	0.74
Residual volume (ml)	36 ± 23	50 ± 14	0.06	35 ± 16	48 ± 14	0.05
Residual volume (%)	26 ± 12	29 ± 8	0.33	24 ± 6	25 ± 4	0.66
Systolic peak kinetic energy						
Total (µJ)	5,261 ± 1,222	5,621 ± 1,649	0.61	5,365 ± 2,066	6,340 ± 3,075	0.25
– index_EDV_ (µJ/ml)	41 ± 13	35 ± 8	0.26	38 ± 11	33 ± 13	0.05
Direct flow (µJ)	2,902 ± 1,185	3,451 ± 1,694	0.42	3,012 ± 1,348	4,023 ± 2,315	0.10
– index_DF_ (µJ/ml)	76 ± 17	83 ± 23	0.51	88 ± 27	83 ± 30	0.52
Residual volume (µJ)	262 ± 205	362 ± 396	0.29	208 ± 104	286 ± 156	0.31
– index_Res_ (µJ/ml)	7 ± 2	6 ± 5	0.70	6 ± 4	6 ± 5	0.99
Early (E) diastolic peak kinetic energy						
Total (µJ)	2,855 ± 965	1,814 ± 833	0.04	1,953 ± 1,102	1,428 ± 678	0.26
– index_EDV_ (µJ/ml)	21 ± 5	11 ± 5	<0.01	14 ± 8	7 ± 2	0.08
Direct flow (µJ)	1,303 ± 553	746 ± 466	<0.01	866 ± 531	555 ± 286	0.27
– index_DF_ (µJ/ml)	36 ± 12	17 ± 7	<0.01	25 ± 12	12 ± 3	0.06
Residual volume (µJ)	429 ± 432	412 ± 314	0.81	167 ± 85	215 ± 117	0.32
– index_Res_ (µJ/ml)	10 ± 4	7 ± 3	0.01	8 ± 1	4 ± 1	0.34
Late (A) diastolic peak kinetic energy						
Total (µJ)	–	3,005 ± 1,559	–	–	2,005 ± 755	–
– index_EDV_ (µJ/ml)	–	19 ± 10	–	–	11 ± 4	–
Direct flow (µJ)	–	1,186 ± 1,000	–	–	809 ± 394	–
– index_DF_ (µJ/ml)	–	26 ± 22	–	–	16 ± 5	–
Residual volume (µJ)	–	183 ± 118	–	–	194 ± 80	–
– index_Res_ (µJ/ml)	–	5 ± 3	–	–	4 ± 1	–

Mean ± SD are shown for intraventricular measurements of patients with cardiovascular disease (*n* = 6) from both 4D sequences which were prospectively (4D_prosp_) and retrospectively gated (4D_retro_). Global kinetic energies are indexed to end-diastolic volume (EDV) obtained from 4D analysis, while the direct flow (DF) and residual volume (Res) are indexed to the respective component volume.

A, late diastole (atrial contribution); E, early diastole; KE, kinetic energy.

## Discussion

**Figure 5:**
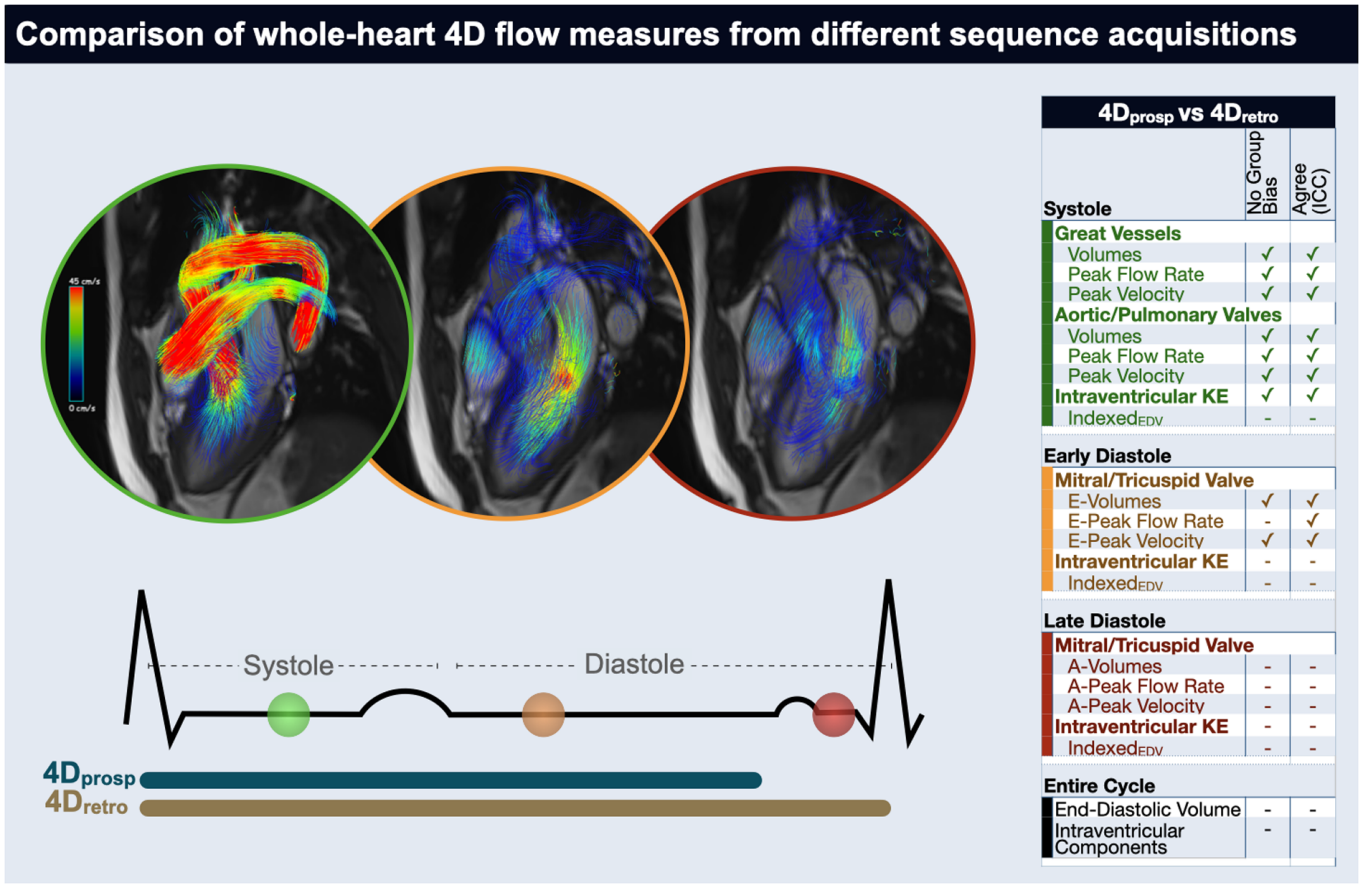
Take-home figure. In a direct comparison between 4D flow acquired by prospective (4D_prosp_) or retrospective (4D_retro_) gating in 32 controls and 6 cardiovascular patients, measurements primarily occurring during systole of the great vessels, semilunar valves and both left and right ventricles did not differ between acquisitions. Additionally, a majority of data dependent on early-diastole was consistent, however measurements occurring in late diastole or reliant on the entire-cardiac cycle were not similar between 4D_prosp_ and 4D_retro_ acquisitions. ✓indicates no significant difference was observed between the gating strategies (first column) measurements (*p* > 0.05) and that there was a good agreement (intraclass correlation coefficient >0.75, second column).

In a direct comparison of whole-heart 4D quantification, two different 4D flow sequences, captured with prospective (4D_prosp_) and retrospective (4D_retro_) gating were both acquired in 32 controls and six cardiovascular patients. Measurements primarily occurring during systole of the great vessels, semilunar valves and both left and right ventricles did not differ between acquisition types and neither did the majority of early diastolic quantifications. However, measurements occurring in late diastole or those reliant on the entire-cardiac cycle were not similar between 4D_prosp_ and 4D_retro_ acquisitions leading to a discrepancy of total and flow component volumes along with kinetic energy diastolic assessments (Figure 5). While multiple parameters that differ between the two sequence variants can play a role in the discrepancy of the quantifications, we suspect that due to the nature of the similarities and differences of the measurements, the largest impact most likely comes from the different gating techniques.

### Systolic function measures of the aorta and aortic valve

Many cardiovascular imaging exams will quantify the aortic root or ascending aorta to measure systolic output of the left heart. In our results, we observed that both sequences yielded the same results for ascending aortic flow measurements and pulse wave velocity of the entire thoracic aorta, and these measures fit within reference ranges defined by 2D CMR imaging. While the aorta often takes the spotlight, 4D flow CMR has been used for pulmonary artery assessments and we observed similar flow rates and velocities in the main pulmonary artery. Total effective volume of the main pulmonary artery was slightly underestimated by 3 ml by the 4D_prosp_. The clinical relevance of this volume may be small, but prospective gating can pose problems if flow through the desired plane occurs very early in systole while the sequence is waiting for the R-wave or still processing the ECG trigger. This has been highlighted for pulse wave velocity assessments, where calculations may be reliant on the initial upslope of the flow curve (29), while another group reported in a multi-vendor, multi-site study that prospective gating underestimated aortic flow (17). However, in the latter study, acquisitions were obtained weeks apart, and not within the same exam as in our study where we expect minimal hemodynamic differences. One could speculate that in very proximal planes which will receive blood early in the cardiac cycle, forward or effective volume may be missed if there is any delay in acquisition, but this can be distinguished by assessment of the beginning of the flow curve. If a delay is present, this shouldn't impact the peak flow rates and velocities in most cases as the peaks generally occur about 15%–25% into the cardiac cycle, or 3–6 phases based on temporal resolution and heart rate. Our measurements from both 4D_prosp_ and 4D_retro_ acquisitions are in line with what other groups have reported and validated against other techniques. Aortic valve peak velocities from 4D flow have been reported to match those observed with echocardiography (30), while pulmonary artery 4D flow was shown to correlate with RV function and mean pulmonary arterial pressures, quantified during right heart catheterization (31, 32). Retrospective imaging may also play a role in pathologies to observe abnormal hemodynamics in the semilunar valves and arteries occurring late in the cardiac cycle, such as abnormal retrograde flow, valve regurgitations, or slow flow through a false lumen in aortic dissections. Hurd et al. found good agreement between prospectively and retrospectively gated sequences, when comparing velocities in the carotid bifurcation of five patients, with an advantage in detecting retrograde flow in the retrospective sequence, due to higher temporal coverage (33). We didn't specifically address abnormalities as the majority of our cohort were young healthy controls, but in Figure 4 of the patient with mild aortic valve disease, light regurgitation during diastole was quantified in the ascending aorta and the aortic valve by both sequences.

### Diastology assessed by mitral and tricuspid valve function

The presence of poor diastolic filling can represent chronically impaired lusitropy and ventricular stiffness, but diastolic function can also be impaired temporarily, especially in altered hemodynamic states such as during intensive care and in perioperative environments (34–37). 4D flow CMR has already been used to quantify peak E-wave velocities and flow rates through both the mitral and tricuspid valve (3, 7, 38). In our analysis we observed the same volume and peak velocity during early diastole with the 4D_prosp_ and 4D_retro_ for both the mitral and tricuspid valve with good to excellent agreement. Peak E-flow rate also yielded a good agreement between gating techniques (all ICC > 0.80), yet the 4D_retro_ systematically underestimated the peak E-flow rate by 11% and 14% for the mitral valve and tricuspid valve respectively. We did not compare our 4D findings to conventional doppler echocardiography, but Assadi et al. found comparable measures between peak E- and A-velocities with 4D flow in heart failure patients, especially for those in sinus rhythm (3). It has also been reported for the tricuspid valve, in patients with congenital heart disease and pulmonary hypertension, quantification of total effective volume through the tricuspid valve, measured by 4D flow, was reproducible and agreed with echocardiography quantification of effective volume through the pulmonary valve (39). The benefit of 4D flow in combination with valve tracking software, is that planes and contours can be defined individually in any direction per phase rather than relying on a static plane, which is especially important for tracking the changing angulation and motion of the tricuspid valve.

As detailed further in the Supplementary material, the A-wave typically occurs in the final 82%–96% of the cardiac cycle. However, late diastolic (A-wave) measures were often not present or complete in the majority of 4D_prosp_ acquisitions, which acquired on average 86% of the cardiac cycle. This lower temporal coverage is due to image planning, where an acquisition window shorter than the RR interval is manually defined by the imaging technician to allow for acquisition of the navigator. The imaging technician also may shorten the acquisition window to ensure it does not extend into the next heartbeat, otherwise data would only be collected every second heartbeat and thus double acquisition time. This supports the 4D flow recommendations to capture the entire cycle with retrospective gating. A-wave measures are important for E/A ratios, another key component for grading diastolic function, and they are important for calculating total mitral and tricuspid effective volume (40). Especially with increasing age, or in patients with primary and secondary diastolic dysfunction, or even a temporary increase in systemic resistance, a significant portion of diastolic inflow can occur in the final phases of the cardiac cycle (6, 41). This is depicted in Figure 4, where peak A-flow rate and A-velocity are significantly higher than the E-wave. Importantly, the acquisition of the final inflow into the ventricle will play a vital role for quantification of ventricular hemodynamics. In the future it will be interesting to relate transmitral E velocity and flow to the corresponding ventricular myocardial distension using CMR, similar to the echocardiographic marker E/e', which is also an excellent marker for LV end-diastolic pressure (40).

### Intraventricular measures

Currently there are a wide variety of techniques used to assess intraventricular 4D flow, including analysis of KE, flow components, vortices, and visual assessments. Intraventricular KE has been investigated in a variety of pathologies including ischemic heart disease, valvular disease and heart failure (9, 42). We observed the global peak systolic KE of both ventricles was consistent between sequences. However, a significantly lower early diastolic peak was quantified from 4D_retro_ in comparison to 4D_prosp_. A possible explanation is that in resting conditions, diastolic timing will change with heart-rate fluctuations more profoundly than systole. This can lead to distortions or temporal smoothing among phases if there are beat-to-beat variations. Additionally, misinterpretation of ECG-Signals, such as triggering of T-waves instead of only R-waves during the acquisition, can lead to incorrect binning of the data to cardiac time frames with the 4D_retro_, distorting the signal as well, although this was not specifically quantified in our study, while with prospective gating these false triggers are ignored.

Intraventricular components and total volumes were also generally not consistent between the 4D_prosp_ and 4D_retro_, with the best agreement observed for the percentage of direct flow components. Direct flow has also been reported as the most reliable parameter in a test-retest study (16). Importantly quantification of both KE and intraventricular components are reliant on the area detected as ventricular lumen by the software and physiologically larger ventricles are associated with higher LV and RV KE (14). This poses a problem with prospective sequences, where the ventricle is not yet at or near full volume, 86% (our median acquisition time) into the cardiac cycle. Consequently, total volumes, and volumes of the components were significantly lower with the 4D_prosp_. Although excellent reader-reliability (6, 43) of intraventricular components and KE has been shown within studies, values differ when comparing reports between groups. For example, a review by Ashkir et al. compared eight publications quantifying intraventricular components in healthy volunteers, reporting mean direct flow varied from 21% to 58% and residual volume from 7% to 33% (9). Thus, limitations of the software and varying analysis techniques likely need to be considered before comparing intraventricular findings.

### Differences in 4D sequences

To investigate different gating techniques, two different sequences, both of which are commonly implemented into imaging exams, were needed. This is because our conventional k*-*t GRAPPA sequence was limited to only prospective gating, and the introduction of the compressed sensing product sequence allowed for a choice in gating. However, parameters unique to the sequences may have the potential to influence agreement as well such as acceleration factors (Table 1) (18). Especially with the advancement of acceleration factors, compressed sensing techniques are ideal for saving time during the CMR exam and faster sequences are likely to be chosen by imaging sites if the option is available. Even with our two sequences, the compressed sensing with retrospective gating required only 60% of the acquisition time. The comparison between acceleration factors has been investigated by various groups. Aortic velocities and flows can be underestimated with both compressed sensing and k-t GRAPPA, but the used acceleration factors in both sequences have been shown to provide reliable flow quantification in comparison to conventional parallel imaging (18, 25, 44). We expect these influences to be small as the k-t GRAPPA sequence we used already had a higher acceleration factor (undersampling *R* = 5, R_net _= 4.3), in comparison to the *R* = 7.6 of the compressed sensing, and the compressed sensing is also based on k-t acceleration. In a publication investigating different 4D sequences on a Philips scanner, Blanken et al. compared 4D flow measurements of the four cardiac valves between a pseudo-spiral 4D flow MRI with prospective undersampling in multiple dimensions and echo planar imaging-accelerated 4D flow (13). Both sequences were retrospective-ECG gated, and they reported that flow, peak velocities and E/A ratios were preserved up to an undersampling factor of 9. These measurements also were similar to the findings of our healthy controls. Similarly, we did not routinely observe discrepancies in systolic and early diastolic flows and velocities, however, we observed discrepancies with the loss of late-diastolic flow. Therefore, it appears these differences are more influenced by the different acquisition windows from the gating type than the sequence type. Nevertheless, the additional variation in sequences may play a role in the disagreement, and further investigation is needed to assess how large of an impact this is, especially for intraventricular measurements. To isolate if discrepancies in measures are solely based on gating techniques, a future study should run the same sequence twice with both gating techniques. This was not possible with our k*-*t GRAPPA 4D_prosp_ which was limited to only prospective triggering, but this could be performed in the future with the compressed sensing sequence which allows for a choice of prospective or retrospective gating. Importantly, the goal of this study was not to assess if one technique is better than the other, rather it was to assess if similar findings are obtained and thus validate if data can be combined within a study or used interchangeably, independent on the technician's choice of gating or if a contemporary sequence was used.

### Research and clinical implications

Because 4D flow is a rapidly developing area, there are a variety of sequences in use. Especially as advancements in acceleration allow for faster acquisition during exams, it is likely imaging sites will adapt to using the updated sequences once available. Therefore, our goal was to investigate if two commonly used sequences were comparable, allowing for future merging of data in large research studies, or comparison of measurements to normal values or between patient cohorts. In particular, we suspected any discrepancies were primarily influenced by the choice of ECG gating. Consensus statements often suggest retrospective gating to capture the hemodynamics of the entire cardiac cycle, as prospective gating is often deemed inferior throughout the field (21, 45). Yet, there are minimal to no studies focusing on how gating may impact measurements and thus minimal evidence to support these consensus statements. Our data confirm that retrospective gating should be used over prospective gating for whole-heart 4D flow acquisitions concerning the acquisition of full diastolic measurements and diastolic-dependent quantifications. What we can also interpolate from our data, is that if retrospective gating is not a possibility, partial data from prospectively gated acquisitions can still be assessed, as quantifications are comparable for the majority of systolic and early diastolic measures, but not for assessments of the entire cardiac cycle. Therefore, this study provides evidence for the current 4D flow consensus statements concerning gating selection for temporal coverage. Moreover, this comparison is important as there may not always be the option to choose retrospective gating. This could be due to limited sequence availability on a scanner, when imaging was performed (e.g., in the case of longitudinal studies or multicentre studies), or if the imaging technician determined prospective triggering was more appropriate. For example, prospective triggering may be selected when the RR interval varies from beat to beat, such as when dealing with arrhythmias or poor triggering. The consensus statements also recommend that prospective gating may be useful in these conditions (21). It is important to clarify that our analysis compares the two types of sequences to each other and does not compare if one is better than the other in comparison to an external reference. A key advantage of this study is both datasets were acquired during the same CMR exam and heart rate did not differ between acquisitions, thus hemodynamic and volume status are not expected to have changed, which otherwise could have a confounding impact on diastolic measurements. Thus, if combining data with different gating techniques, these findings imply systolic measures of the aorta and pulmonary artery, semilunar valves and systolic KE are consistent between the two acquisitions and many early diastolic markers can be compared with caution. If retrospective gating is not used, a reader can compare the mismatch in effective flow volume between the semilunar and atrioventricular valves, and mismatch in 4D flow derived EDV from their prospectively-triggered acquisition to standard volume analysis to assess if late diastolic and ventricular findings may be influenced by the missing phases of the cardiac cycle, especially in the case of expected high late diastolic contribution to ventricular filling. Despite the inherent difficulties that can be present when investigating the right heart, especially with 2D imaging, measurements from the RV and the tricuspid valve, had similar agreement as observed in the LV. This supports the use of whole-heart 4D analysis. Our data were acquired with an ECG trigger, and not pulse triggering. If the latter were selected, then diastole would be acquired with loss of systolic information.

### Limitations

We did not assess the diagnostic ability of the techniques to discriminate groups or to detect cardiovascular dysfunction such as dissections, regurgitation, and vortices, nor did we acquire 2D phase-contrast flow images as a comparator. Moreover, analysis was only quantitative. The image quality of the different sequences and the ability to visually depict abnormalities was not investigated. We used two sequences available at our institution, which both were only single-VENC. Multi-VENC acquisitions may improve quantification further by better accounting for both high velocities observed in the arteries and low velocities in the apexes. Additionally, we only assessed two versions of 4D flow, findings may differ with other sequences. Finally, this analysis utilized a single software vendor. With this software, masks had to be redefined for each analysis which may impact quantification. Analysis was blinded between the 4D_prosp_ and 4D_retro,_ thus the independent placement of analysis planes by the reader may impact measurements. Comparisons between vendor analysis should still be investigated, as software is rapidly developing.

## Conclusion

The use of 4D flow CMR is rapidly expanding, and so are applications for investigating the vessels, valves and cardiac chambers. We directly compared two 4D acquisitions, a routine 4D sequence with prospective (4D_prosp_) gating to a contemporary compressed sensing 4D flow with retrospective (4D_retro_) gating and observed that most systolic and many early diastolic markers were not dependent on the choice of sequence. However late diastolic measurements and many intraventricular parameters differed between the 4D_prosp_ and 4D_retro_ acquisitions, and this difference is likely due to gating strategy. Therefore, these findings confirm and provide evidence supporting consensus statements that retrospective gating should be the first choice for acquiring 4D flow datasets when quantifying the entire cardiac cycle. If the entire cardiac cycle is not acquired due to prospective gating, systolic and early diastolic parameters should be compatible. Yet incomplete acquisition of the cardiac cycle has a significant impact on mitral and tricuspid A-wave dynamics, and intraventricular quantifications should be compared with caution.

## Data Availability

The raw data supporting the conclusions of this article will be made available by the authors, without undue reservation.
