# Supplementary material for: Direct comparison of whole heart quantifications between different retrospective and prospective gated 4D flow CMR acquisitions
Source: Front Cardiovasc Med. 2024 Jul 31;11:1411752. doi: 10.3389/fcvm.2024.1411752 (PMC11322094; doi:10.3389/fcvm.2024.1411752)
Supplement: Supplementary file 1 [file Datasheet1.pdf]

SUPPLEMENTARY MATERIAL

Direct comparison of whole heart quantifications between different retrospective and prospective gated 4D flow CMR acquisitions

TABLE OF CONTENTS

SUPPLEMENTARY RESULTS: TIMING OF SYSTOLIC AND DIASTOLIC TRANSVALVULAR FLOW 2

SUPPLEMENTARY TABLE 1: PARTICIPANT CHARACTERISTICS 3

Supplementary Results: Timing of Systolic and Diastolic Transvalvular Flow

Flow through the aortic valve started at the beginning of the cycle and ended at 37±5% into the cardiac cycle (353±33ms). Blood flow through the mitral valve started shortly thereafter, with early (E) diastolic flow lasting from 38±4% (368±46ms) to 68+6% (666±88ms) of the cardiac cycle. Typically, after a period of diastasis the late diastolic flow (A) occurred between 82±7 (811±187ms) and 96±6% (934±187ms) of the cardiac cycle.

For the right heart, similar timepoints were observed with blood flow through the pulmonary valve lasting from the start of the cycle until 38±5% (364±30ms), early diastolic flow through the tricuspid valve occurring between 38±5% (362±40) and 70±9% (678±103ms), and late diastolic flow between 81±8% (804±196ms) and 95±5% (926±191ms).

Supplementary Table 1: Participant Characteristics

	Controls (n=32)	Patients (n=6)
Baseline		
Sex (males)	17 (53%)	4 (66%)
Age, range (years)	27 [19-41]	70 [60-78]
Body Mass Index (kg/m^2)	23.1±2.9	27.0±3.5
Left Ventricular Function		
EDV (ml)	167±30	167±33
EDV index (ml/kg^2)	89±10	86±12
Ejection Fraction (%)	59±5	57±11
Mass index (g/kg^2)	53±9	52±7
Cardiac index (L/min/m^2)	3.3±0.7	3.3±0.7
Right Ventricular Function		
EDV (ml)	197±42	177±44
EDV index (ml/kg^2)	101±15	90±19
Ejection Fraction (%)	44±6	47±9

Frequency (percentage) or mean±SD are displayed of patient characteristics and functional analysis measured from standard cines. Full short-axis stacks of the right heart were only available in n=20 healthy controls. EDV: End-diastolic volume.
